# *Ixodes ricinus* ticks have a functional association with *Midichloria mitochondrii*

**DOI:** 10.3389/fcimb.2022.1081666

**Published:** 2023-01-09

**Authors:** Melina Garcia Guizzo, Tereza Hatalová, Helena Frantová, Ludek Zurek, Petr Kopáček, Jan Perner

**Affiliations:** ^1^ Institute of Parasitology, Biology Centre of the Czech Academy of Sciences, České Budějovice, Czechia; ^2^ Faculty of Science, University of South Bohemia, Ceske Budejovice, Czechia; ^3^ CEITEC, University of Veterinary Sciences, Brno, Czechia; ^4^ Department of Microbiology, Nutrition and Dietetics/CINeZ, Czech University of Life Sciences, Prague, Czechia; ^5^ Department of Chemistry and Biochemistry, Mendel University, Brno, Czechia

**Keywords:** *Ixodes ricinus*, ticks, symbionts, *Midichloria*, membrane feeding, tetracycline, mitochondria

## Abstract

In addition to being vectors of pathogenic bacteria, ticks also harbor intracellular bacteria that associate with ticks over generations, aka symbionts. The biological significance of such bacterial symbiosis has been described in several tick species but its function in *Ixodes ricinus* is not understood. We have previously shown that *I. ricinus* ticks are primarily inhabited by a single species of symbiont, *Midichloria mitochondrii*, an intracellular bacterium that resides and reproduces mainly in the mitochondria of ovaries of fully engorged *I. ricinus* females. To study the functional integration of *M. mitochondrii* into the biology of *I. ricinus*, an *M. mitochondrii*-depleted model of *I. ricinus* ticks was sought. Various techniques have been described in the literature to achieve dysbiosed or apo-symbiotic ticks with various degrees of success. To address the lack of a standardized experimental procedure for the production of apo-symbiotic ticks, we present here an approach utilizing the *ex vivo* membrane blood feeding system. In order to deplete *M. mitochondrii* from ovaries, we supplemented dietary blood with tetracycline. We noted, however, that the use of tetracycline caused immediate toxicity in ticks, caused by impairment of mitochondrial proteosynthesis. To overcome the tetracycline-mediated off-target effect, we established a protocol that leads to the production of an apo-symbiotic strain of *I. ricinus*, which can be sustained in subsequent generations. In two generations following tetracycline administration and tetracycline-mediated symbiont reduction, *M. mitochondrii* was gradually eliminated from the lineage. Larvae hatched from eggs laid by such *M. mitochondrii*-free females repeatedly performed poorly during blood-feeding, while the nymphs and adults performed similarly to controls. These data indicate that *M. mitochondrii* represents an integral component of tick ovarian tissue, and when absent, results in the formation of substandard larvae with reduced capacity to blood-feed.

## 1 Highlights

- *ex vivo* membrane feeding of ticks is a powerful tool for the production of apo-symbiotic ticks
- *Midichloria mitochondrii*-free *I. ricinus* females produce larvae that are less successful during blood-feeding
- use of tetracycline shows off-target effects on proteosynthesis in tick mitochondria

## 2 Introduction

The co-existence of microbiota and eukaryotic hosts underpins much of the complexity of the natural world and experimental studies demonstrate that these associations confer benefits to the hosting eukaryote (Douglas, 2014). Arthropods, too, commonly display stable associations with microbial symbionts and rarely exist as symbiont-independent entities (Hammer et al., 2019). The wide distribution of microbial-arthropod associations often results in a nutritional advantage (synthesis or digestion) to the host (Douglas, 2009) and symbiont-dependent species have often evolved specialized tissues or organs with micro-niches where the microbiota flourish (Douglas, 2020). Such microbial association is often essential only at a specific point in ontogeny, e.g. *Daphnia* egg (Mushegian et al., 2018).

Experimental axenic or dysbiosed variants of blood-feeding arthropod species have been instrumental in understanding the functional integration of microbes into the physiology of their hosts (Narasimhan et al., 2014; Romoli et al., 2021; Gilliland et al., 2022). While mosquitoes are inhabited by a substantial amount of bacterial biomass, its functional significance in mosquito physiology and development appears to be minor, if any, under the conditions examined (Steven et al., 2021). Ticks, on the other hand, associate with very few, probably transient, microbes in the digestive tract (Ross et al., 2018; Guizzo et al., 2020) but generally harbor ovary-resident bacterial symbionts (Bonnet et al., 2017; Buysse et al., 2021). The functional significance of the microbiota in tick physiology and development has typically been investigated using antibiotic treatment (Duron et al., 2018; Zhong et al., 2021).

*Midichloria mitochondrii* is the dominant tick-associated bacterium (Epis et al., 2008), and was originally reported to be associated with 100% of *Ixodes ricnus* wild-caught females (Lo et al., 2006), although it now appears that the symbiont species differs geographically across Europe (Krawczyk et al., 2022). *Midichloria mitochondrii* occupies primarily the mitochondrial inter-membrane space in ovarian cells (Buysse and Duron, 2018), where it multiplies upon the tick engorging a blood meal (Sassera et al., 2008). Transmission electron microscopy images and mathematical simulation, however, did not support predatory behavior of *M. mitochondrii* on *I. ricinus* mitochondria (Comandatore et al., 2021), contrasting with the original assumption (Sacchi et al., 2004). If *M. mitochondrii* is not predatory in nature, its function or benefit might be sought. In *I. ricinus*, however, there are no established protocols for the production of apo-symbiotic ticks depleted of *M. mitochondrii*, which would be key for obtaining functional data on *M. mitochondrii* - *I. ricinus* symbiosis.

To bridge this gap, we have established such a protocol using artificial *ex vivo* blood membrane feeding of *I. ricinus*. We show that oral antibiotic administration is clearly superior to micro-injection, being able to profoundly reduce levels of *M. mitochondrii* in generation zero. In generations subsequent to tetracycline administration, a complete elimination of *M. mitochondrii* was achieved in the lineage but the larvae produced from these apo-symbiotic females have a reduced fitness, pointing towards a functional significance of *M. mitochondrii*.

## 3 Materials and methods

### 3.1 Tick rearing

Wild-caught unfed *I. ricinus* females were collected by flagging around Ceske Budejovice, Czech Republic. All tick life stages were maintained under controlled conditions (temperature: 24°C and humidity: 95%) in the rearing facility at the Institute of Parasitology, Biology Centre of the Czech Academy of Sciences. All laboratory animals were treated in accordance with the Animal Protection Law of the Czech Republic No. 246/1992 Sb., ethics approval No. 25/2018. The study was approved by the Institute of Parasitology, Biology Centre CAS and Central Committee for Animal Welfare, Czech Republic (Protocol No. 1/2015).

### 3.2 Generation of *Midichloria mitochondrii*-free ticks by *ex vivo* membrane blood feeding

Wild-caught *Ixodes ricinus* adult females were fed defibrinated bovine blood *via* an *ex vivo* membrane feeding system (Kröber and Guerin, 2007). Every 12 hours for a period of 10 days, the feeding chambers were washed with a multipurpose disinfectant, 0.5% (w/v) Virkon S (Chemours), followed by a sterile solution NaCl (0.9%) and placed in clean cell culture plate-wells filled with 3.1 mL of fresh blood supplemented with 50 µg/mL or 5 µg/mL tetracycline (Sigma-Aldrich: T3383). The antibiotic low- and high-dosage is very similar to the previously published amounts of antibiotics used for blood meal supplementation (Oliver et al., 2021). These working concentrations were obtained by 1000 × dilution from their stock solutions prepared in DMSO. The blood was freshly supplemented with every 12h blood exchange regime. Individual *I. ricinus* females were thus dosed with 100 µmol or 10 µmol of tetracycline, respectively, assuming a 1mL uptake of blood meal. Fully fed females that spontaneously detached from the membrane were randomly placed into two groups: in one of these, the females had their ovaries dissected out for DNA isolation, and in the other, the females were placed individually into glass vials kept at 24°C and 95% humidity to allow egg laying and subsequent hatching of larvae. The first generation was initiated by placing 15 mg of unfed *I. ricinus* larvae from *M. mitochondrii*-reduced (from *I. ricinus* females dosed with 100 µmol of tetracycline) and control females on guinea pigs, and the second generation was initiated by placing 20 mg of unfed *I. ricinus* larvae from *M. mitochondrii*-free and control females. For comparison, wild-caught *I. ricinus* females were fed naturally on laboratory guinea pigs, and freshly engorged females were micro-injected into the haemoceol with 350 nL of tetracycline, which was solubilized in MilliQ H_2_O as 150 mg/mL, 15 mg/mL, or 1.5 mg/mL stock solutions. Individual *I. ricinus* females were thus dosed with 112 nmol, 11.2 nmol, and 1.12 nmol of tetracycline, respectively.

### 3.3 Evaluation of tick fitness by natural feeding on a host

Larvae, nymphs and adult ticks from control and *Midichloria*-free groups were naturally fed to repletion on guinea pigs in separated compartments. Control ticks used in this study originated from the progeny of wild-caught adult females ticks naturally fed on lab-reared hosts. Guinea pigs were examined once a day for the presence of fully fed ticks. Fully fed larvae and nymphs were placed in glass vials to molt to the following life stage. Fully engorged females were allowed to perform egg laying and subsequent hatching of larvae. Larval engorgement was calculated by the number of larvae that fully engorged and dropped off the host. Nymphal and adult female engorgement successes were evaluated by the percentage of ticks that fed until repletion, detaching spontaneously from the host. Nymphal and adult molting successes were calculated by the percentage of nymphs and adults molted from engorged larva and nymphs, respectively.

### 3.4 Genomic DNA isolation and quantification of *Midichloria mitochondrii*

Total DNA was isolated from either dissected ovary (from individual adult females under sterile PBS), eggs (n = 30), or the whole tick at different life stages using the PowerSoil Kit (Mobio) according to the manufacturer’s instructions. DNA was eluted in 100 μL with concentrations ranging from 1.8 ng/μL to 14.7 ng/μL. *Midichloria mitochondrii* was quantified as described by (Sassera et al., 2008). The real-time PCR reaction mixture contained 2 μL of DNA, 10 μL of SYBR green (Roche, 4913914001), 1 μL of each primer (10 pmol) (F: 5’CTTGAGAGCAGAACCACCTA 3’; R: 5’CAAGCTCTGCC GAAATATCTT 3’) and DNA-free water (Top-Bio, Praha, Czech Republic) to 20 μL. The *I. ricinus* single-copy gene *elongation factor-1α* (*ef-1α*; ID: GU074769.1*)* was used as a reference for data normalization and also as a control for the DNA isolation procedure (F: 5’ACGAGGCTCTGACGGAAG 3’; R: 5’CACGACGCAACTCCTTCAC 3’). The reaction was carried out in a QuantStudio 6 qPCR instrument (Applied Biosystems) with 50 cycles at 95 °CC (10 s), 60 °CC (10 s), and 72 °CC (10 s) following an initial denaturation at 95 °CC (10 min). The samples were considered free of *M. mitochondrii* when no specific amplification of the bacterium could be detected within 50 cycles.

### 3.5 Medium preparation and cell cultures

The *I. ricinus* embryonic cell line (IRE/CTVM19) (Bell-Sakyi et al., 2007) was propagated in Leibovitz L-15 medium (Biosera, LM-L1050), supplemented with 20% (v/v) human serum (Sigma- Aldrich, H4522), Tryptose Phosphate Broth (10% w/v) (Sigma-Aldrich, T8159), Antibiotic and Antimycotic (1% w/v; Biosera XC-A4110), and L-glutamine (1% w/v; Biowest, X0550). To test the sensitivity of tick mitochondrial proteosynthesis to individual antibiotics, 6mL cell cultures (6 × 10^5^ cells/mL media) were transferred to a medium without the antibiotic and antimycotic supplement and were instead supplemented with 400 µg/mL (final concentration) of tetracycline, chloramphenicol (Sigma-Aldrich: C0378), or gentamicin (Sigma-Aldrich: G1914) according to (Moullan et al., 2015). Cells were cultured for 72 hours and then centrifuged at 160 × g for 5 min, rinsed with 1× phosphate-buffered saline (PBS), and again centrifuged at 160 × g for 5 min. Cellular pellets were homogenized and proteins were solubilized in 500 µL of 1× PBS pH 7.4, 1mM EDTA, 1µM E-64 (Merck: E3132) by three freeze-thaw cycles in liquid nitrogen. The homogenate was clarified by centrifuging at 10 000 × g, 10 min, 4°C. The consequent soluble fraction was discarded and the membrane protein-enriched pellets were solubilized in reducing Laemmli buffer.

### 3.6 SDS-PAGE and western blotting

Ovaries from fully engorged *I. ricinus* females were dissected under DEPC-treated PBS. Three individual ovaries were used for RNA extraction and three individual ovaries were homogenized and used for protein solubilization as described above. Protein homogenates of tick ovarian tissue or embryonic cell line were adjusted to the equal protein load and separated by reducing SDS-PAGE in 4–20% Stain-free gels (BioRad: 4568095). Protein separation was visualized in the stain-free mode and proteins were transferred to 0.2µm pore PVDF membrane (BioRad: 1704272) for Western blotting. The membrane was blocked in non-fat milk suspended in PBS-Tween for 1 hour at room temperature. Primary antibodies anti-ATP5A (Abcam: ab14748) and anti-MTCO1(Thermo Fisher Scientific: 459600) were diluted in PBS-Tween 1:500 and 1:1000, respectively, and incubated with the membrane overnight. Immunodetection was performed by anti-mouse peroxidase-conjugated immunoglobulin (Sigma-Aldrich: A9044), diluted 1: 10 000 in PBS-Tween, and the signal was visualized by Clarity™ Western ECL substrate (BioRad: 170-5061) using ChemiDoc (BioRad).

### 3.7 RNA extraction, cDNA synthesis, dsRNA synthesis, and RNAi evaluation by RT-qPCR

RNA was extracted from ovaries using a NucleoSpinRNA II kit (Macherey-Nagel, Germany). The quality of RNA was checked by gel electrophoresis on an agarose gel (1%, w/vol), and stored at -80 °C prior to cDNA synthesis. cDNA preparations were made from 0.5 μg of total RNA in independent triplicates using the Transcriptor High-Fidelity cDNA Synthesis Kit (Roche Diagnostics, Germany). The cDNA served as templates for subsequent quantitative expression analyses by RT-qPCR. Samples were analyzed by a LightCycler 480 (Roche) using Fast Start Universal SYBR Green Master Kit (Roche). Relative expressions were calculated using the ΔΔCt method (Livak and Schmittgen, 2001). The expression profiles were normalized to *I. ricinus* elongation factor 1α (*ef-1α*; ID: GU074769.1). Primers used were: *ir-atp5A* (F: 5’GCTACCTGGACAAGTTGGACC 3’, R: 5’CTTCTGTGTCGACCTGATGTG 3’), *ir-mtco1* (F: 5’GTTTTTGATTATTACCCCCTTCTTT 3’, R: 5’ACTGTTCATCCAGTTCCTGCC 3’).

The target sequence for RNAi was obtained by PCR using the following primers: *ir-atp5A* (F: 5’ atgggccCGACAGGCAGACTGGTAAG 3’, R: 5’ attctaGAGAGATCGTCGTAGATGATAAG 3’), *ir-mtco1* (F: 5’atgggccCTCTTCCTGTTCTTGCTGGAG 3’, R: 5’attctagaCTCGAGCTTACTTTACAGCTGC 3’). dsRNA of *ir-atp5A* (GenBank: GIYG01000932.1), *ir-mtco1* (GIXL01030069.1), or *gfp* (green fluorescent protein) used as a control were synthesized using the MEGAscript T7 transcription kit (Ambion, Lithuania) according to the previously described protocol (Hajdusek et al., 2009; Perner et al., 2018). This resulted in a synthesis and hybridization of dsRNA products of 309 bp (*ir-atp5a*) and 331 bp (*ir-mtco1*). *I. ricinus* females were injected into the haemocoel through to the coxae with these dsRNAs (0.5 μL; 3 μg/μL) or control *gfp* dsRNA (0.5 μL; 3 μg/μL) using a glass capilary of the outer dimater of 20 – 25 µm and a microinjector (Narishige), allowed to rest for one day and then fed naturally for 7 days on guinea pigs. The efficiency of RNA-mediated silencing of *ir-atp5A* gene expression was verified both at the transcript level by RT-qPCR and at the protein level by Western blot analysis using αATP5A antibodies. In this way, we validated specificity of commercial antibodies against non-tick immunogen to recognize its tick homologue.

### 3.8 Statistical analysis

Statistical analyses were performed using GraphPad Prism software (version 8). Differences in DNA amplicon abundances, tick engorged weights, or weights of egg clutches were calculated using the t-test. The values were considered statistically significant with p<0.05 denoted as *, p<0.005 denoted as **, and p<0.0001 denoted as ****.

## 4 Results

### 4.1 Reduction of *Midichloria mitochondrii* abundance in *Ixodes ricinus* ovaries is achieved by feeding ticks tetracycline during *ex vivo* membrane blood feeding

**Figure 1:**
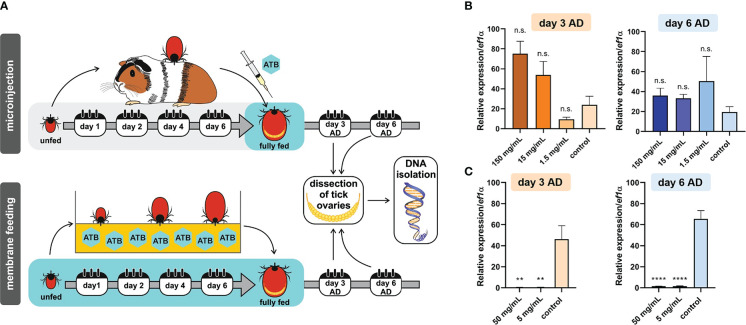
**(A)** Scheme of tetracycline antibiotics (ATB) administration (microinjection or *ex vivo* membrane feeding) and a timeline of DNA extraction from tick ovaries after tick detachment (AD). **(B)** Microinjection. Real-time PCR on DNA extracts from individual tick ovaries determining the levels of *Mitochondria mitochondrii* upon tetracycline microinjection into hemolymph of *Ixodes ricinus* females fully engorged *in vivo*. **(C)** Membrane feeding. Real-time PCR on DNA extracts from individual tick ovaries determining the levels of *M. mitochondrii* upon tetracycline supplementation of blood meal during *ex vivo* membrane feeding of *I. ricinus* ticks. **(B, C)** Stock concentrations of tetracycline are shown. Mean and SEM are shown, **(B)** n = 3 ticks, **(C)** n ≥ 9 ticks. T-test analysis: **p < 0.005; ****p < 0.0001; n.s., not significant.

We previously determined that ovaries of *I. ricinus* females were virtually exclusively colonized by a single bacterial species, *M. mitochondrii* (Guizzo et al., 2020). As a proxy for the effectiveness of bacterial clearance from fully engorged *I. ricinus* females, we determined the relative amounts of *M. mitochondrii* in tick ovaries (**Figure 1A**). First, we performed a one-time microinjection of fully engorged *I. ricinus* females with tetracycline in a concentration series, and quantified levels of *M. mitochondrii* three and six days after feeding (and injection). At both time-points, there was no reduction in levels of *M. mitochondrii* in tick ovaries (**Figure 1B**). To increase the overall dosage of tetracycline achieved in ticks, we performed *ex vivo* tick membrane blood feeding supplemented with two different concentrations (50 µg/mL and 5 µg/mL; final concentrations) of tetracycline. With this approach, we achieved a significant reduction in *M. mitochondrii* levels in tick ovaries, three and six days after engorgement (**Figure 1C**). We noted, however, that at higher concentration of tetracycline, these *I. ricinus* females were less successful than control ticks in their capacity to fully engorge (see below).

### 4.2 Tetracycline inhibits proteosynthesis in tick mitochondria

**Figure 2:**
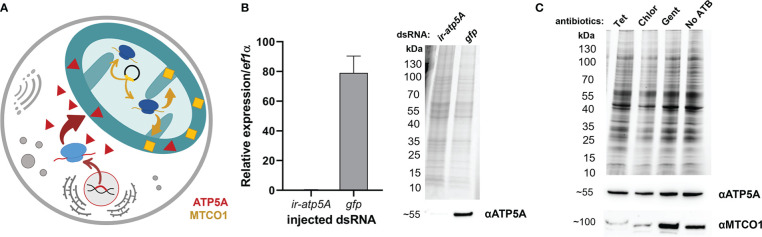
**(A)** A scheme depicting two distinct genomic origins of mitochondrial proteins, one from the nuclear gene (red) and one from mitochondria (yellow). **(B)** Validation of anti-ATP5A antibody by transcript knockdown of *ir-atp5a* using RNAi. RT-qPCR on cDNA from ovaries (left) and Western blotting on protein homogenates of ovaries (right) from fully engorged *I. ricinus* females are shown. **(C)** Western blot analysis detecting two mitochondrial proteins ATP synthase subunit alpha (ATP5A) and Mitochondrially Encoded Cytochrome C Oxidase I (MTCO1) in a homogenate of tick cells. Tet, Tetracyclin; Chlor, Chloramphenicol; Gent, Gentamicin; No ATB, no antibiotics. **(B, C)** Proteins were separated using reducing SDS-PAGE and visualized by the stain-free system (BioRad). Below, western blot analyses are shown using commercial antibodies against ATP5A (αATP5A) and MTCO1 (αMTCO1).

In order to evaluate the potential of off-target tetracycline activity to inhibit mitochondrial proteosynthesis, as reported in other eukaryotes (Moullan et al., 2015), we designed a Western blot assay detecting two mitochondrial proteins; one encoded by the nuclear genome and the other by the mitochondrial genome. ATP synthase F1 subunit alpha (ATP5A) is encoded by the nuclear genome, is translated in the cytosol, and only then is transported into the mitochondria, while the Mitochondrially Encoded Cytochrome C Oxidase I (MTCO1) is encoded in the genome of mitochondria, where it is also synthesized by the mitochondrial-based translation apparatus (**Figure 2A**). Upon specificity validation of the commercial antibodies against non-tick homologues (**Figure 2B**), we noted that tetracycline treatment reduced the levels of MTCO1 in tick mitochondria while the levels of ATP5A remained unchanged (**Figure 2C**). These data show that tetracycline inhibited tick mitochondrial proteosynthesis and thus may elicit activity affecting tick fitness. Using RNAi, we could only validate specificity against ATP5A, as we could not achieve RNAi silencing for the mitochondrial protein MTCO1 (**Supplementary Figure S1**). Instead, we confirmed the anti-MTCO1 specificity of the commercial antibodies using chloramphenicol as a specific inhibitor of mitochondrial proteosynthesis (Richter et al., 2019) (**Figure 2C**).

### 4.3 An *ex vivo* membrane platform for tick blood feeding as a tool for generation of a *Midichloria mitochondrii*-free strain of *I. ricinus*

**Figure 3:**
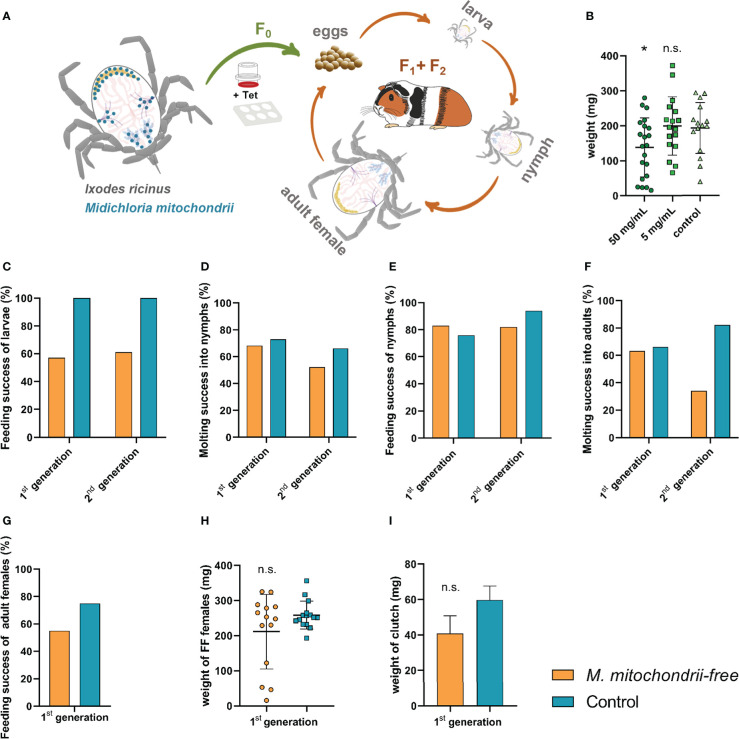
Effect of the absence of *Midichloria mitochondrii* on tick engorgement and development across two generations. **(A)** A scheme depicting the production of *M. mitochondrii*-free strain of *I. ricinus* ticks. The *ex vivo* membrane feeding is indicated by a feeder and a 6-well plate; Tet, tetracycline. The green arrow indicates a reduction in *M. mitochondrii* levels in generation zero, while the orange arrows indicate the propagation of apo-symbiotic *I.ricinus* lineage upon *M. mitochondrii* elimination. The blue dots in the cartoon of unfed *I. ricinus* female (top left) indicate tissue tropism of *M. midichloria*, according to (Olivieri et al., 2019). **(B)** Weights of fully engorged *I. ricinus* females upon blood feeding tetracycline-supplemented blood meal *via ex vivo* membrane feeding. Stock concentrations of tetracycline are shown. Mean and SEM are shown, n ≥ 15; *p < 0.05; n.s., not significant. **(C)** Success feeding rate of *M. mitochondrii*-free larvae in two generations subsequent to adult *I. ricinus* clearance. **(D)** Molting success of these larvae into nymphs. **(E)** Feeding success rate of *M. mitochondrii*-free nymphs in two generations subsequent to adult *I. ricinus* clearance. **(F)** Molting success of these nymphs into adults. **(G)** Feeding success rate of *M. mitochondrii*-free adults in a generation subsequent to adult *I. ricinus* clearance. **(H)** Weights of these *M. mitochondrii*-free *I. ricinus* adult females upon *in vivo* engorgement. **(I)** Weights of egg clutches laid by 1^st^ generation *M. mitochondrii*-free *I. ricinus* females. **(B, H, I)** Mean and SEM are shown. Original numbers to the bar graphs are shown in **Supplementary Table S1**.

In order to avoid possible immediate mitochondrial toxicity of tetracycline, we set out to produce a strain of *I. ricinus* ticks that would be distanced in time from the administered tetracycline (**Figure 3A**). *Ixodes ricinus* females were fed tetracycline-supplemented blood meal (**Figure 3B**) *via* the *ex vivo* membrane feeding system, and the larvae were used as starting progenies for the *M. mitochondrii*-free strain. These progenies were monitored for two following generations and assessed for their capacity to blood-feed, develop, and reproduce. In a generation subsequent to adult bacterial clearance by feeding, larvae exhibited a reduced capacity to engorge *in vivo* on laboratory animals. Engorgement was reduced to 57% in the first generation and to 61% in the second generation (**Figure 3C**). The failure of the cohort to normally blood-feed was manifested in profoundly reduced numbers of larvae reaching engorgement in 48 hours (**Supplementary Figure S2**). Larvae that fed successfully then developed into nymphs with a rate similar to that of the control group (**Figure 3D**). Furthermore, nymphs also displayed no deviation from controls (**Figures 3E, F**). While we observed an impairment of females during the clearance feeding itself (**Figure 3B**) with a longer time to engorge and lower engorged weight, we did not observe any reduced capacity of adult females to blood-feed in the generation after clearance (**Figures 3G, H**), strongly suggesting an off-target effect of the higher dose of tetracycline. The egg mass produced by *M. mitochondrii*-free and control groups of the second generation of *I. ricinus* females were not statistically significant either (**Figure 3I**). The extent of reduction of *M. mitochondrii* levels through the two generations cycle was monitored by qPCR (**Supplementary Figure S3**). These data clearly indicated that the use of tetracycline in the *ex vivo* blood feeding system led to an immediate reduction in *M. mitochondrii* in this parenting generation (F_0_), but eventually reached complete elimination (i.e. beyond the limits of real time PCR detection) of *M. mitochondrii* in the following generation of *I. ricinus* females (F_1_) (**Supplementary Figure S3**). These data show a clear correlation between the partial failure of larvae to blood-feed and the *M. mitochondrii*-free status of their female ancestor. The causative link, however, still remains to be unambiguously determined.

## 5 Discussion

The increasing number of studies on the diversity and quantity of tick microbiota starts creating a distinct sub-field of tick biology. Functional studies stemming from experimental models of ticks with manipulated inner bacterial ecosystems only start to appear, keeping pace with other symbiotic systems (see below). In mosquito research, two main protocols have been developed describing the development of axenic mosquitoes: (i) the use of disinfectants on oviposited eggs (Coon et al., 2014; Correa et al., 2018) and (ii) using a transient colonization/decolonization approach (Romoli et al., 2021). It has been demonstrated that axenic mosquitoes do not show any developmental retardation, reach the same size as controls (Correa et al., 2018; Romoli et al., 2021), and can therefore be reared under axenic conditions for multiple generations, similarly to mice and fruit flies (Steven et al., 2021).

In this study, we used two different administrations of tetracycline, the antibiotic that has been used in previous reports of successful elimination of other Gram-negative intracellular symbionts in several tick species (Zhong et al., 2007; Guizzo et al., 2017; Zhong et al., 2021). While we, and others (Ninio et al., 2015), failed to reduce *M. mitochondrii* loads in *I. ricinus* ticks through tetracycline micro-injection, both microinjection as well as membrane feeding of *I. scapularis* ticks with ciprofloxacin was effective in depleting or eliminating *Rickettsia buchneri* (Oliver et al., 2021). We thus believe that the membrane feeding platform, which is available for multiple species of hard ticks (Gonzalez et al., 2021), represents a universal and powerful tool for the production of apo-symbiotic ticks.

*Midichloria mitochondrii* is the dominant bacterium in ovaries of fully engorged *I. ricinus* females (Guizzo et al., 2020) and it is vertically transmitted from the parent to the offspring (Sassera et al., 2006). Therefore, a reduction in this bacterium in the tick ovaries enabled us to produce a following generation of *I. ricinus* in which *M. mitochondrii* was eliminated, while minimizing the confounding off-target effect of tetracycline. This allowed us to determine the effect of tick endogenous bacteria on development and fitness of its host *I. ricinus*. We found that larval feeding success was equally negatively impacted in both the first and second generations of bacteria-free *I. ricinus* ticks. These data are in line with those of the obligate symbionts *Coxiella* sp. in *Rhipicephalus microplus* (Guizzo et al., 2017) and *Francisella* sp. in *Ornithodoros moubata* (Duron et al., 2018). In these cases, bacterial elimination caused an interruption in the regular course of tick development in the short-term, where it impaired the feeding of nymphs of *O. moubata*, or it stopped feeding and development at the nymphal stage of *R. microplus*. Larval feeding success was also inhibited in *Rhipicephalus sanguineu*s when *Coxiella* sp. was eliminated (Ben-Yosef et al., 2020). In *R. microplus*, the comparative transcriptomic analysis of *Coxiella*-free nymphs revealed a substantial under-expression of tick transcripts involved in their blood-feeding capacity (Guizzo et al., 2022).

The functional aspects of the bacterial microbiota of blood-feeding arthropods, integrating their metabolic products with their physiology, has started to emerge, as reviewed by (Song et al., 2022). The metabolic interaction between bacteria and ticks, with a distinct impact on development and/or physiology, seems to be very species-specific (Duron et al., 2018; Zhong et al., 2021). In addition, the methods used across the studies are not unified, which prevents us formulating general statements on this topic. Overall, the administration of antibiotics has been applied to manipulate bacterial levels and/or diversity in order to study the role of bacteria in tick biology (Narasimhan et al., 2014; Guizzo et al., 2017; Zhang et al., 2017; Duron et al., 2018; Zhong et al., 2021). These reports mainly comprise microinjection into the tick haemocoel, administration of antibiotics into vertebrate hosts, or *ex vivo* membrane blood feeding as tools to effectively deliver antibiotics into ticks and thus disturb the microbial community. A wide range of negative impacts on tick fitness has been reported as a result of disturbance in the abundance of *Coxiella* sp. and *Francisella* sp. in their tick hosts (Zhong et al., 2007; Guizzo et al., 2017; Zhang et al., 2017; Duron et al., 2018; Ben-Yosef et al., 2020), thus indicating a functional interaction between maternally-inherited bacteria and their tick hosts. Having said that, we are far from a full appreciation of the functional derivation of bacterial symbiosis and integration of bacterial metabolites into physiology of ticks. For a successful endosymbiosis to occur in nature, a flux of carbon and nitrogen in the form of amino acids (Esser et al., 2019), which represent the “molecular currency” providing the housing eukaryote a benefit. The pilot study in identifying such molecular currency in tick host symbiosis was recently published, demonstrating the key involvement of *Coxiella*-produced chorismate in the metabolism of amino acids (tryptophan) of the *Haemophysalis longicornis* ticks (Zhong et al., 2021).

Altogether, our data suggest a functional integration of bacterial symbiosis, most likely with *M. mitochondrii*, into developmental and physiological processes of *I. ricinus* ticks. With *M. mitochondrii* being unculturable, it is technically not feasible to rescue the apo-symbiotic *I. ricinus* females with the bacterium to restore the physiology of maternal ovaries and thus the production of fully fit larvae. This represents a clear limitation of our study. The fact that we cannot link the observed phenotype of slow larval feeding and the overall lower feeding success rate of larvae (from tetracycline-treated ancestors) with *M. mitochondrii* leaves open the possibility of the significance of another bacterial species or bacterial mass regardless of the bacterial taxon. Having said that, the maternal inheritance of *M. mitochondrii*, may qualify this bacterium as a prime bacterial entity candidate, facilitating the formation of more successful larvae. Another interpretation of our data might be that the functional association is not key during embryogenesis and larval formation, but that the functional association with bacteria occurs in tick larvae themselves. On another technical note, the ability of tetracycline to inhibit proteosynthesis in tick mitochondria warrants caution. We advise to only use tetracycline as a means of producing apo-symbiotic strains of ticks but discourage using it as an anti-bacterial agent for phenotypisation of the organism immediately after receiving tetracycline treatment. We argue that the trans-stadial and trans-generational distance between the use of tetracycline and phenotypisation legitimizes its use.

## Data availability statement

The datasets presented in this study can be found in online repositories. The names of the repository/repositories and accession number(s) can be found in the article/**Supplementary material**.

## Author contributions

MG, conceived the study, overseen data analysis, perfomed experiments, and wrote a first manuscript draft. TH, performed experiments, analyzed data, and prepared figures. HF, performed experiments. LZ, edited manuscript and funding acquisition PK, edited manuscript, funding acquisition, and data analysis. JP, conceptualized the work, wrote the final manuscript version, and funding acquisition. All authors contributed to the article and approved the submitted version.
