# Supplementary material for: Ixodes ricinus ticks have a functional association with Midichloria mitochondrii
Source: Front Cell Infect Microbiol. 2023 Jan 9;12:1081666. doi: 10.3389/fcimb.2022.1081666 (PMC9868949; doi:10.3389/fcimb.2022.1081666)
Supplement: Supplementary file 1 [file DataSheet_1.pdf]

Suuplementary Table S1

	Generation	Engorged larvae; individuals and (percentages)	nymphs molted from engorged larvae	engorged nymphs from nymphs molted	molted adults from engorged nymphs	engorged females / molted females	egg laying / engorged females	larvae hatching / clutches of eggs
Control	1st	321 (100%); start: 15 mg unfed larvae	222 / 306 = 72.5%	163 / 212 = 77%	101 (43 females + 58 males) / 154 = 66%	15 / 20 = 75%	9 / 12 = 75%	8 / 9 = 89%
M. mitochondrii-free	1st	183 (183 / 321 = **57%**); start: 15 mg unfed larvae	115 / 168 = 68%	84 / 101 = 83%	47 (24 females + 23 males) / 75 = 63%	11 / 20 = 55%	6 / 8 = 75%	6 / 6 = 100%
Control	2nd	454 (100%); start: 20 mg unfed larvae	291 / 439 = 66%	102 / 109 = 94%	76 (34 females + 42 males) / 93 = 82%	not available	not available	not available
M. mitochondrii-free	2nd	279 (279 / 454 = **61%**); start: 20 mg unfed larvae	138 / 264 = 52%	89 / 109 = 82%	27 (12 females + 15 males) / 80 = 34%	not available	not available	not available

* note that some individuals are missing in the trans-stadial evaluation; these represent natural die off or ticks that were taken for DNA extractions.
