# Supplementary material for: Ixodes ricinus ticks have a functional association with Midichloria mitochondrii
Source: Front Cell Infect Microbiol. 2023 Jan 9;12:1081666. doi: 10.3389/fcimb.2022.1081666 (PMC9868949; doi:10.3389/fcimb.2022.1081666)
Supplement: Supplementary file 2 [file Image_1.pdf]

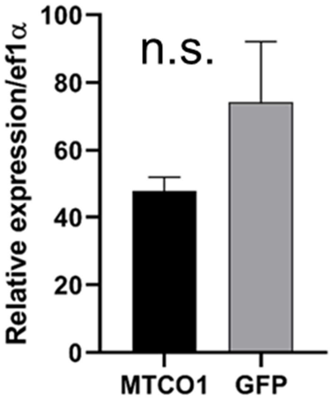

Supplementary Figure S1. RT-qPCR validation of RNAi-silencing of *Ixodes ricinus* Mitochondrially Encoded Cytochrome c Oxidase I (MTCO1) in ovaries of fully engorged *Ixodes ricinus* females. Mean and SEM are shown, n = 3 (ovaries from three different females); n.s., not significant.
