# Supplementary material for: Ixodes ricinus ticks have a functional association with Midichloria mitochondrii
Source: Front Cell Infect Microbiol. 2023 Jan 9;12:1081666. doi: 10.3389/fcimb.2022.1081666 (PMC9868949; doi:10.3389/fcimb.2022.1081666)
Supplement: Supplementary file 3 [file Image_2.pdf]

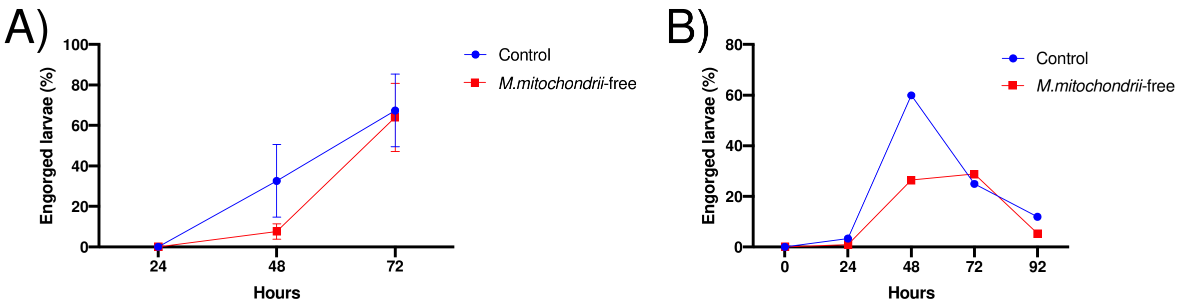

Supplementary Figure S2: Kinetics of successful blood-feeding in Midichloria mitochondrii-free Ixodes ricinus larvae in first (A) and second (B) generations. A: The engorgement rate was expressed as the mean with SD of two biological replicates.
