# Supplementary material for: Ixodes ricinus ticks have a functional association with Midichloria mitochondrii
Source: Front Cell Infect Microbiol. 2023 Jan 9;12:1081666. doi: 10.3389/fcimb.2022.1081666 (PMC9868949; doi:10.3389/fcimb.2022.1081666)
Supplement: Supplementary file 4 [file Image_3.pdf]

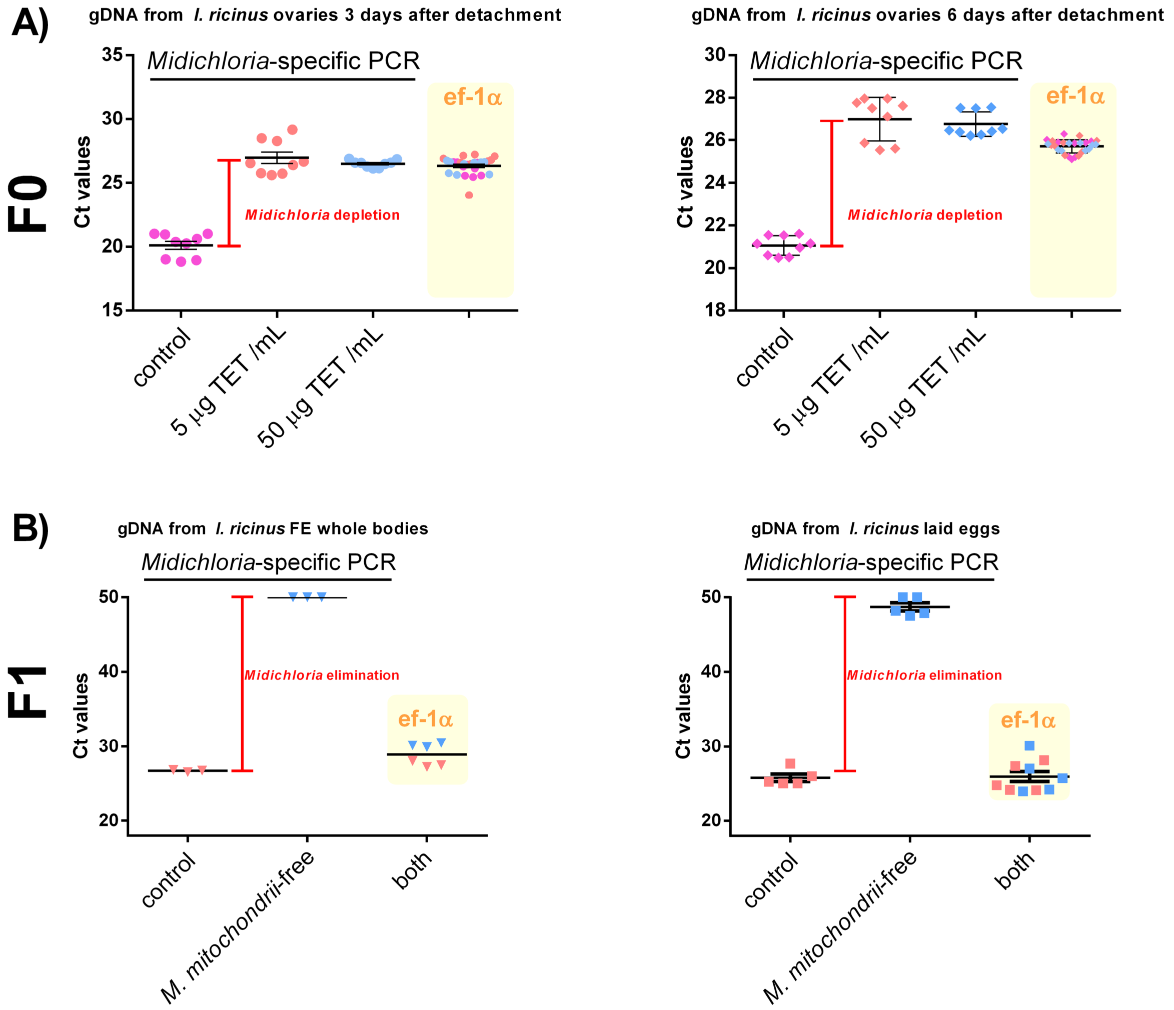

Supplementary Figure S3. *Midichloria mitochondrii* levels determined by real-time PCR. **A)** *M. mitochondrii* levels determined in ovaries of engorged *Ixodes ricinus* females, 3 days after detachment (on left) and 6 days after detachment (on right). Levels of *M. mitochondrii* were evaluated based upon *ex vivo* membrane blood feeding of ticks fed blood supplemented with 50 µg or 5 µg of tetracycline (TET) in 1 ml of blood, i.e. generation zero. **B)** *M. mitochondrii* levels determined in first generation *M. mitochondrii-free* fully engorged (FE) females and in their laid eggs. *elongation factor* (*ef-1α*) served as a target gene in the tick genome. Each dot represents a Ct value from three technical replicates of three – five biological replicates. Means and SDs are shown.
